# Coordination of matrix attachment and ATP-dependent chromatin remodeling regulate auxin biosynthesis and *Arabidopsis* hypocotyl elongation

**DOI:** 10.1371/journal.pone.0181804

**Published:** 2017-07-26

**Authors:** Kyounghee Lee, Pil Joon Seo

**Affiliations:** Department of Biological Sciences, Sungkyunkwan University, Suwon, Republic of Korea; Instituto de Biologia Molecular y Celular de Plantas, SPAIN

## Abstract

Hypocotyl elongation is extensively controlled by hormone signaling networks. In particular, auxin metabolism and signaling play key roles in light-dependent hypocotyl growth. The nuclear matrix facilitates organization of DNA within the nucleus, and dynamic interactions between nuclear matrix and DNA are related to gene regulation. Conserved scaffold/matrix attachment regions (S/MARs) are anchored to the nuclear matrix by the AT-HOOK MOTIF CONTAINING NUCLEAR LOCALIZED (AHL) proteins in *Arabidopsis*. Here, we found that ESCAROLA (ESC)/AHL27 and SUPPRESSOR OF PHYTOCHROME B-4 #3 (SOB3)/AHL29 redundantly regulate auxin biosynthesis in the control of hypocotyl elongation. The light-inducible AHL proteins bind directly to an S/MAR region of the *YUCCA 9* (*YUC9*) promoter and suppress its expression to inhibit hypocotyl growth in light-grown seedlings. In addition, they recruit the SWI2/SNF2-RELATED 1 (SWR1) complex and promote exchange of H2A with the histone variant H2A.Z at the *YUC9* locus to further elaborately control auxin biosynthesis. Consistent with these results, the long hypocotyl phenotypes of light-grown genetic mutants of the AHLs and H2A.Z-exchanging components were suppressed by potent chemical inhibitors of auxin transport and YUC enzymes. These results suggest that the coordination of matrix attachment and chromatin modification underlies auxin biosynthesis in light-dependent hypocotyl growth.

## Introduction

Plant development is regulated depending on light conditions. The *Arabidopsis* hypocotyl is a remarkable system for studying genetic contributions to light-dependent plant development: light inhibits hypocotyl growth and etiolation of seedlings (photomorphogenesis), whereas plants grown in dark conditions exhibit long hypocotyls, apical hook formation, and lack of chloroplast development (skotomorphogenesis) [1]. Light-modulated hypocotyl growth requires versatile hormone pathways, and consistently, many metabolic and signaling components of plant hormones have been identified as crucial regulators for hypocotyl growth [2–5].

Auxin metabolism and signal transduction are closely associated with hypocotyl elongation in *Arabidopsis*. Indole-3-acetic acid (IAA) is mainly synthesized by tryptophan (Trp)-dependent pathways [6,7]. In particular, YUCCAs (YUCs), flavin-containing monooxygenases, are key catalytic enzymes that oxidize indole-3–pyruvic acid, which is converted from tryptophan by TRYPTOPHAN AMINOTRANSFERASE OF ARABIDOPSIS 1 (TAA1)/SHADE AVOIDANCE 3 (SAV3) [8–10]. Notably, auxin-overproducing plants display long hypocotyls under light conditions [11,12], whereas auxin-deficient mutants, such as *sav3-2* and *cyp79B2 cyp79B3* mutants, exhibit reduced hypocotyl length [11,13].

Auxin is sensed by a co-receptor complex consisting of an F-box protein from the TRANSPORT INHIBITOR RESPONSE 1/AUXIN SIGNALING F-BOX PROTEIN (TIR1/AFB) family and a member of the AUXIN/INDOLE ACETIC ACID (AUX/IAA) family that inhibits transcriptional activity of AUXIN RESPONSE FACTOR (ARF) transcription factors [14]. In the absence of auxin, AUX/IAA proteins bind to ARFs to repress transcriptional activity, together with the co-repressor TOPLESS (TPL) [15]. In the presence of auxin, the SKP1–Cul1–F-box (SCF)^TIR1/AFB^ coreceptor stimulates ubiquitination and protein turnover of the AUX/IAA proteins to activate ARFs [15]. Consistently, auxin signaling mutants, including *auxin resistant2-1* (*axr2-1*), *non-phototropic hypocotyl4* (*nph4*)/*arf19* and *short hypocotyl2-2* (*shy2-2*) exhibit reduced hypocotyl length in darkness [16–18].

The nuclear matrix is an insoluble filamentous network structure inside of a cell nucleus that facilitates the exquisite organization of DNA within the nucleus. Intimate interactions between the nuclear matrix and DNA are highly ordered [19], and scaffold/matrix attachment regions (S/MARs), which are conserved DNA elements in eukaryotes, are specifically involved in anchoring chromatin to the nuclear matrix [20]. These regions are recognizable as AT-rich sequences that are 300–5000 bp in length [21,22].

The organization of S/MARs with the nuclear matrix is dynamically regulated and contributes to complicated regulation of gene expression [23]. In general, intragenic S/MARs show a negative correlation with transcriptional activity [23,24], whereas intergenic S/MARs usually activate expression of adjacent genes [25]. Furthermore, S/MARs are sometimes linked with epigenetic control that also affects gene expression. S/MARs often serve as binding sites for chromatin remodeling factors and facilitate reorganization of chromatin structure [26]. In addition, some S/MAR-binding proteins interact with histone modifiers [27,28], further underscoring the importance of S/MAR-mediated chromatin organization in gene regulation.

A number of S/MAR-binding proteins have been identified in many eukaryotes. A major group of the S/MAR-binding proteins contains two conserved protein domain units, the AT-hook motif and the plant and prokaryote conserved (PPC) domain/domain of unknown function #296 (DUF296) [29]. The *Arabidopsis* genome encodes 29 AT-HOOK-CONTAINING NUCLEAR-LOCALIZED (AHL) proteins, and they are implicated in a variety of plant developmental processes [30–32]. For example, AHL18, AHL22, AHL27/ESCAROLA (ESC)/ORESARA 7 (ORE7), and AHL29/SUPPRESSOR OF PHYTOCHROME B-4 #3 (SOB3) delay flowering possibly by negatively regulating *FLOWERING LOCUS T* (*FT*) expression [30]. In addition, the ESC protein systemically regulates leaf senescence [33]. AHL21 controls *ETTIN* (*ETT*)*/ARF3* expression during reproductive organ differentiation by regulating deposition of H3K9me2 at associated target genes [34].

Notably, ESC and SOB3 function redundantly to regulate hypocotyl elongation. The dominant-negative *sob3-6* allele exhibits long hypocotyl phenotypes in light [31,35], and consistently, the *sob3-4 esc-8* double mutant also displays increased hypocotyl growth [31,35]. The AHL proteins form extensive interactive networks with themselves and other regulatory proteins to facilitate proper plant development [31]. Accumulating evidence addresses that transcriptional control of auxin metabolism and signaling is associated with SOB3-dependent hypocotyl growth [32]. However, a comprehensive view of the signaling networks regulated by ESC and SOB3 remains to be fully defined. Here, we report that ESC and SOB3 mainly regulate auxin biosynthesis by repressing *YUC9* expression in the control of hypocotyl elongation. They bind to a S/MAR in the *YUC9* promoter and facilitate H2A.Z variant exchange to repress *YUC9* expression. These observations propose that the coordination of matrix attachment and chromatin modification underlies auxin biosynthesis in the control of hypocotyl elongation.

## Results

### ESC/AHL27 and SOB3/AHL29 regulate auxin biosynthesis

**Fig 1:**
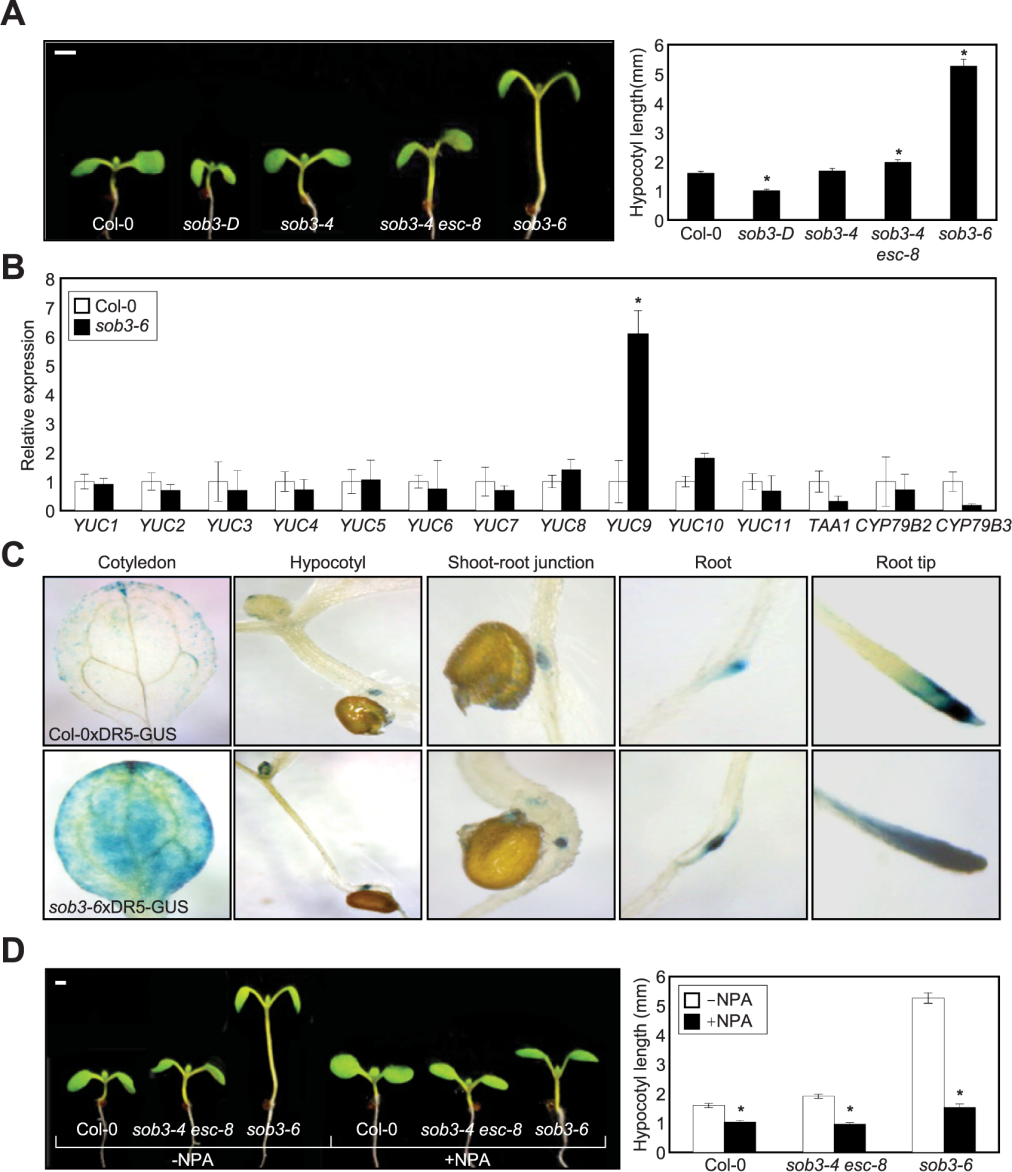
Increased hypocotyl elongation and auxin accumulation in the *sob3-6* mutant. (**A**) Hypocotyl growth of light-grown mutant seedlings. Seeds were germinated and grown for 7 days on vertical plates under long-day (LD) conditions (left panel). Hypocotyl lengths (*n* > 30 in each genotype) were measured using Image J applications (http://rsb.info.nih.gov/ij/). Three biological replicates were averaged and statistically analyzed by two-tailed Student's *t*-test assuming unequal variance. Statistically significant differences between wild-type and mutants are indicated by asterisks (*P < 0.05). Bars indicate standard error of the mean (right panel). (**B**) Transcript accumulation of *YUC*s in *sob3-6*. Transcript accumulation was analyzed by RT–qPCR. The *eIF4a* (At3g13920) gene was used as an internal control. Three biological replicates were averaged and statistically analyzed by two-tailed Student's *t*-test assuming unequal variance (*P < 0.05). (**C**) Enhanced auxin signaling in *sob3-6*. The *pDR5*:*GUS* construct was introduced into wild-type and the *sob3-6* mutant. Seven-day-old seedlings grown under LD conditions were subjected to GUS staining. (**D**) Effects of NPA on hypocotyl elongation of *sob3-6*. Seeds were germinated on MS medium supplemented with 1 uM NPA and incubated for 7 days under LD conditions. Hypocotyl lengths (*n* > 30 in each genotype) were measured using Image J applications (http://rsb.info.nih.gov/ij/). Scale bars, 1 mm.

To investigate the detailed molecular mechanisms underlying AHL-mediated regulation of hypocotyl elongation, we first obtained genetic mutants including *sob3*-*4*, *sob3*-*4 esc*-*8*, *sob3*-*6* and *sob3*-*D*, and measured their hypocotyl length. Consistent with previous reports [31,35], while single mutants did not exhibit phenotypic alterations, the *sob3-4 esc-8* double mutant and dominant-negative *sob3-6* allele displayed long hypocotyl phenotypes in light-grown seedlings (Fig 1A). In contrast, the gain-of-function *sob3*-*D* mutant exhibited a slightly shorter hypocotyl compared with wild-type seedlings (Fig 1A). We also evaluated hypocotyl elongation of mutant seedlings grown in darkness. Notably, hypocotyl length of the *sob3*-*4 esc*-*8* and *sob3-6* mutants was comparable to that of wild-type seedlings, when grown in darkness (S1 Fig). In agreement with the light-dependent phenotypic changes, *ESC* and *SOB3* were significantly induced by light (S2 Fig).

To elucidate the signaling networks underlying S/MAR-regulated hypocotyl growth, we analyzed transcript accumulation of multiple hormone signaling and response components such as *GH3*.*4*, *SMALL AUXIN UPREGULATED RNA 64* (*SAUR64*), *EXPANSIN 1* (*EXP1*), *GIBBERELLIC ACID INSENSITIVE* (*GAI*), *ETHYLENE-INSENSITIVE 3* (*EIN3*) and *ETHYLENE AND SALT INDUCIBLE 1* (*ESE1*). Quantitative real-time RT-PCR (RT-qPCR) analysis revealed that auxin response genes, including *GH3*.*4* and *SAUR64*, were significantly and specifically up-regulated in the *sob3-6* mutant (S3 Fig), suggesting that auxin pathways are under the control of the AHL proteins. When the expression of *PIN-FORMED* (*PIN*) genes encoding auxin efflux carriers was examined, we found that expression of the genes was also increased in the *sob3-6* mutant (S3 Fig). Among the genes involved in auxin metabolism, *YUC9* transcript level was considerably elevated in *sob3-6* (Fig 1B), while other auxin biosynthetic genes were unaffected (Fig 1B). The regulation of *YUC9* by AHLs was relevant in the late stages of hypocotyl elongation (S4 Fig). Transgenic plants overexpressing *YUC9* displayed long hypocotyl phenotype (S5 Fig), which accounts for hypocotyl elongation in the *sob3-6* mutant. Considering the hierarchy of auxin pathways [14], the AHL proteins perhaps control auxin biosynthesis and influence the subsequent auxin signaling cascade.

To verify the roles of ESC and SOB3 in auxin biosynthesis, we genetically crossed the *sob3-6* mutant with *DR5*:*GUS* plants. Histochemical analysis revealed that the GUS expression area was expanded, and GUS strength was enhanced in the *sob3-6* seedlings relative to the wild-type seedlings (Fig 1C). In particular, GUS activity clearly increased in *sob3-6* cotyledons (Fig 1C), which is the primary site of auxin biosynthesis in the seedlings [36]. This is in agreement with the fact that *ESC* and *SOB3* are highly expressed in early seedlings, including cotyledons [35]. In addition, we also analyzed effects of auxin transport inhibitor 1-naphthylphthalamic acid (NPA) on hypocotyl growth of the *sob3*-*4 esc*-*8* and *sob3-6* seedlings. Their long hypocotyl phenotypes in light were suppressed by the exogenous application of NPA (Fig 1D). These observations suggest that the AHL proteins regulate auxin biosynthesis in light-modulated hypocotyl elongation.

### AHLs bind to the *YUC9* promoter

The AHL proteins are likely recruited to cognate DNA regions with AT-rich sequences [37]. We examined whether the ESC and SOB3 proteins bind directly to the auxin biosynthetic genes. Based on public database searches for putative S/MAR regions in the *Arabidopsis* genome (SMARTest, http://www.genomatix.de/), we found that most *YUC* loci contain at least one intragenic S/MAR or intergenic S/MAR near the coding regions. Since the *YUC9* level alone increased in the *sob3-6* seedlings (Fig 1B), we hypothesized that SOB3 and ESC proteins bind to the *YUC9* locus.

**Fig 2:**
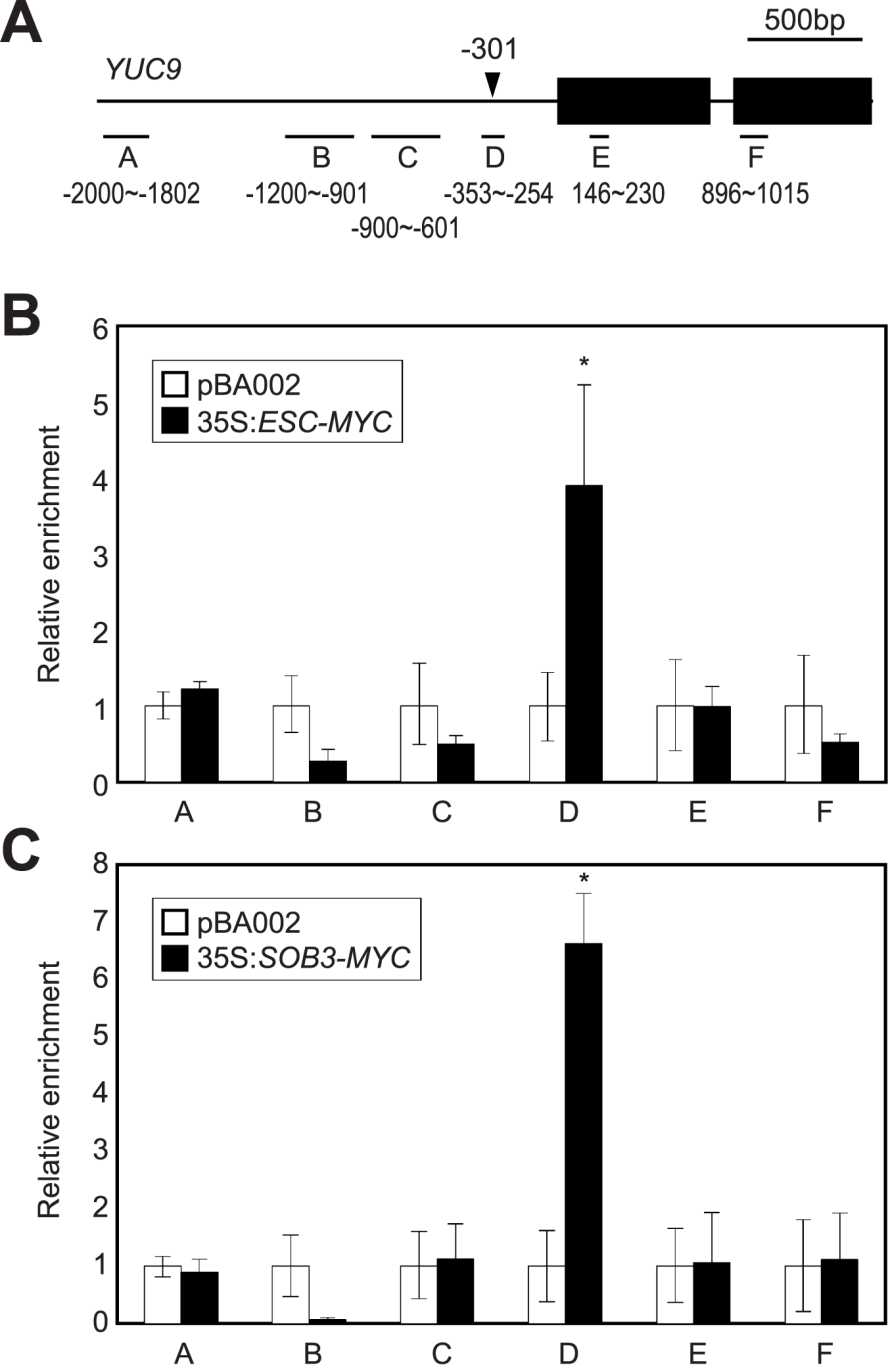
Binding of ESC and SOB3 to the *YUC9* promoter. (**A**) Putative S/MAR region in the *YUC9* promoter. The predicted S/MAR region is marked with an arrowhead. Underbars represent the amplified genomic regions. **(B** and **C**) ChIP assays. Total protein extracts from 35S:*ESC-MYC* (**B**) and 35S:*SOB3-MYC* (**C**) transgenic plants grown for 9 days under LD conditions were immunoprecipitated with an anti-MYC antibody. Fragmented DNA was eluted from the protein-DNA complexes and used for qPCR analysis. Three independent biological replicates were averaged, and the statistical significance of the measurements was determined by two-tailed Student's *t*-test assuming unequal variance (*P < 0.05). Bars indicate the standard error of the mean.

To test this possibility, we produced 35S:*ESC-MYC* and 35S:*SOB3-MYC* transgenic plants and used them to perform chromatin immunoprecipitation (ChIP) assays with anti-MYC antibody. Quantitative PCR (qPCR) analysis using eluted DNA fragments after immunoprecipitation showed that the matrix attachment region D of the *YUC9* promoter was specifically enriched (Fig 2A–2C), while other regions in the *YUC9* locus with no S/MAR were not enriched (Fig 2A–2C). Furthermore, chromatin precipitation in the absence of antibody did not enrich the D region (S6 Fig). In addition, the AHL proteins did not associate with other S/MAR-containing *YUC* loci, except for *YUC8* (S7 Fig). We observed weak association of AHLs to *YUC8* but focused only on *YUC9*, because the binding to the *YUC9* promoter was more obvious in our conditions. These results indicate that ESC and SOB3 mainly bind to the S/MAR of the *YUC9* promoter.

### AHLs stimulate H2A.Z exchange at the *YUC9* promoter

Intergenic S/MARs are frequently associated with gene activation [25]. Considering that ESC and SOB3 suppress the *YUC9* gene by the association with its promoter region, we examined the possibility that additional regulatory schemes may be involved. It was noticeable that some AHL proteins recruit other nuclear proteins, such as chromatin remodeling machineries, to regulate gene expression [27,28]. Several recent reports also provide evidence that S/MAR is associated with epigenetic modifications [38,39].

**Fig 3:**
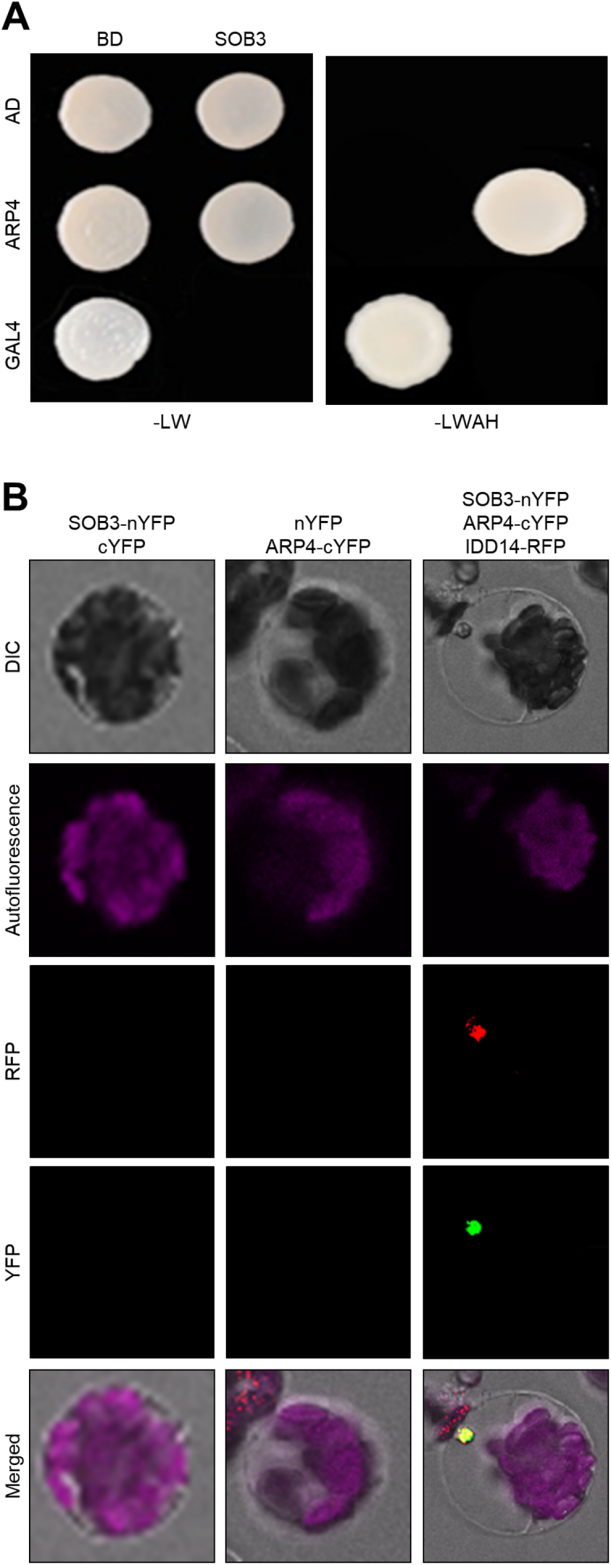
Interaction of SOB3 with the SWR1 component. (**A**) Y2H analysis. Y2H assays were performed with the SOB3 protein fused to the DNA-binding domain (BD) of GAL4 and ARP4 fused with the transcriptional activation domain (AD) of GAL4 for analysis of interactions. Interactions were examined by cell growth on selective media. -LWHA indicates Leu, Trp, His, and Ade drop-out plates. -LW indicates Leu and Trp drop-out plates. GAL4 was used as a positive control. (**B**) BiFC assays. Partial fragments of YFP protein were fused with SOB3 and ARP4. IDD14-RFP was used as a nuclear marker.

To test this possibility, we performed yeast-two-hybrid (Y2H) screening using a prey library composed only of approximately 100 cDNAs encoding chromatin remodelers and chromatin modifiers in *Arabidopsis*. Initial screen showed that the SOB3 protein specifically interacts with ACTIN-RELATED PROTEIN 4 (ARP4), a component of *Arabidopsis* SWI2/SNF2-RELATED 1 (SWR1) complex that catalyzes the ATP-dependent exchange of histone H2A for the H2A.Z variant [40]. To verify this observation, we generated a construct containing full-size ARP4 fused to the GAL4-DNA binding domain (BD) and coexpressed this construct with the GAL4 activation domain (AD)-SOB3. Colony formation on selective medium showed that the yeast cells expressing both the SOB3 and ARP4 proteins grow on nutrient-deficient medium (Fig 3A and S8 Fig), suggesting the physical association of SOB3 with ARP4. In contrast, other SWR1 components did not form a protein complex with SOB3 (S9 Fig).

To provide further support for the *in vivo* interaction of SOB3 with ARP4, we conducted bimolecular fluorescence complementation (BiFC) analysis using *Arabidopsis* protoplasts. We transiently coexpressed SOB3-nYFP and ARP4-cYFP constructs in *Arabidopsis* protoplasts and observed yellow fluorescence mainly in the nucleus (Fig 3B). In addition, coexpression of SOB3-nYFP and another core SWR1 component SERRATED LEAVES AND EARLY FLOWERING (SEF)-cYFP also resulted in yellow fluorescence (S10 Fig), indicating that SOB3 is included in the SWR1 complex through direct interactions with ARP4. We did not observe direct interaction between ARP4 and ESC. It might be plausible that while they interact to each other, interaction strength would be very low.

**Fig 4:**
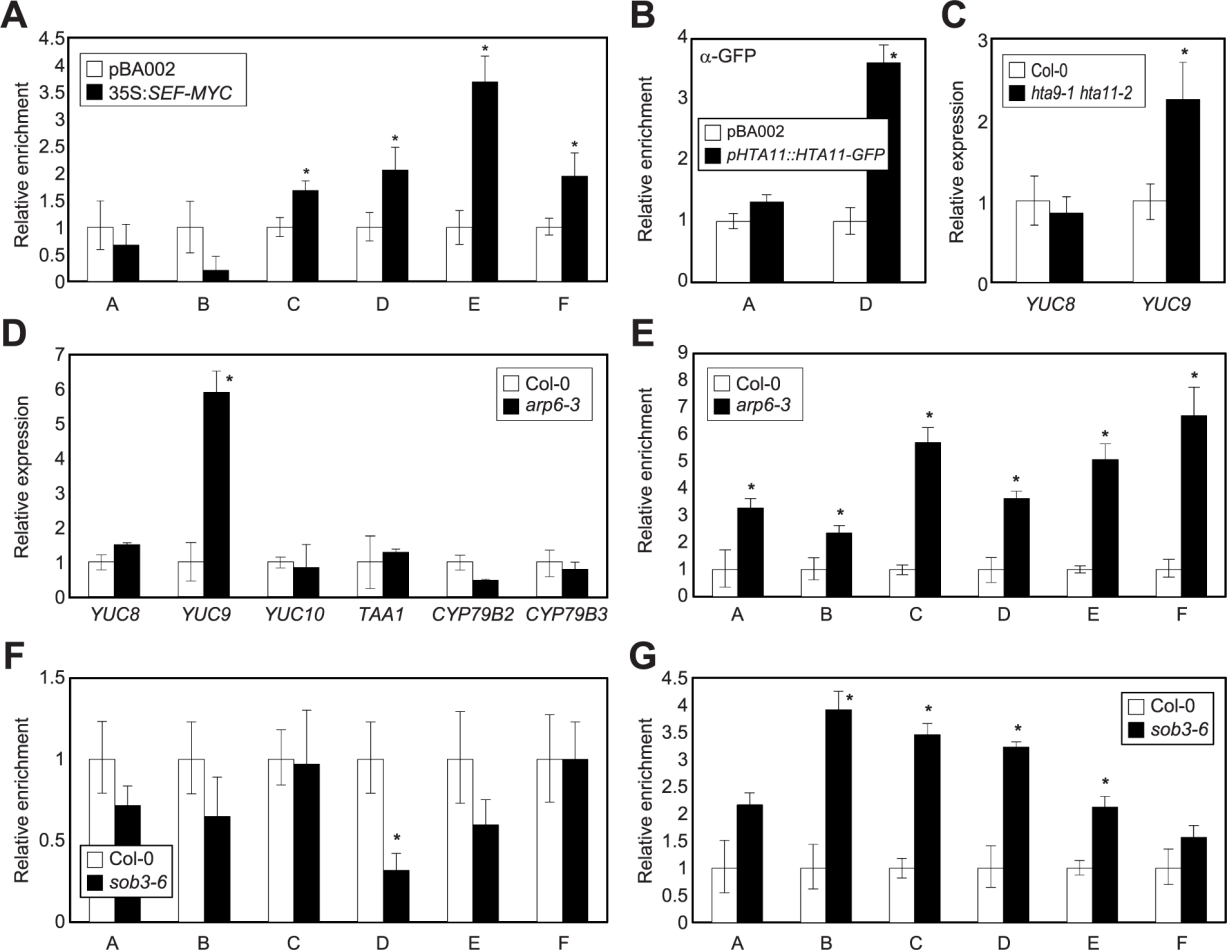
Regulation of *YUC9* expression by the SWR1 complex. (**A**) Binding of SEF to the *YUC9* promoter. Two-week-old 35S:*SEF-MYC* transgenic plants grown under LD conditions were used to conduct ChIP assays. (**B**) H2A.Z deposition in the *YUC9* promoter. Two-week-old *pHTA11*::*HTA11-GFP* transgenic plants grown under LD conditions were used for ChIP analysis with anti-GFP antibody. Eluted DNA was subject to qPCR analysis. (**C** and **D**) Transcript accumulation of *YUC9* in the genetic mutants of H2A.Z exchange. Nine-day-old *hta9-1 hta11-2* (**C**) and *arp6-3* (**D**) mutants grown under LD conditions were used to examine transcript accumulation. Three biological replicates were averaged and statistically analyzed by two-tailed Student's *t*-test assuming unequal variance (*P < 0.05). (**E**) Recruitment of Pol II at the *YUC9* promoter in *arp6-3*. Two-week-old plants grown under LD conditions were used to conduct ChIP assays with an anti-N-terminus of *Arabidopsis* Pol II antibody. (**F**) H2A.Z deposition at the *YUC9* promoter in *sob3-6*. Two-week-old plants grown under LD conditions were used to conduct ChIP assays with an anti-H2A.Z antibody. (**G**) Recruitment of Pol II at the *YUC9* promoter in *sob3-6*. Two-week-old plants grown under LD conditions were used to conduct ChIP assays with an anti-N-terminus of *Arabidopsis* Pol II antibody.

Consistent with our results, the SWR1 complex is known to regulate hypocotyl growth [41]. Genetic mutants of the SWR1 components displayed long hypocotyls in light [41], whereas hypocotyl growth of the mutants was comparable to that of wild-type seedlings in darkness (S11 Fig), similar to the *sob3-6* mutant. These light-dependent hypocotyl phenotypes could be explained by the differential expression of genes encoding SWR1 components in light conditions (S12 Fig). In support of the functional overlap, the key SWR1 component SEF was specifically recruited to regions near the S/MAR of the *YUC9* promoter (Fig 4A), where ESC and SOB3 were targeted (Fig 2). As a consequence, the H2A.Z variant protein HTA11 was indeed deposited in the *YUC9* locus (Fig 4B). We then asked whether transcript accumulation of the *YUC* genes was influenced by genetic mutations in *ARP6*, which is the core component of the SWR1 complex. Notably, the *YUC9* gene was specifically up-regulated in the *arp6-3* and *hta9-1 hta11-2* mutants (Fig 4C and 4D) with higher Pol II recruitment (Fig 4E).

To next investigate whether ESC and SOB3 regulate the incorporation of H2A.Z at the *YUC9* promoter, we performed a ChIP assay with the anti-H2A.Z antibody using wild-type and *sob3-6* mutant plants. qPCR analysis revealed that deposition of H2A.Z at the *YUC9* promoter was impaired in the *sob3-6* mutant (Fig 4F). Consistently, Pol II accessibility was also enhanced in the *sob3-6* mutant (Fig 4G), indicating that the AHL proteins require H2A.Z deposition for the proper control of *YUC9* expression.

### YUC is epistatic to ESC and SOB3 in the control of hypocotyl growth

**Fig 5:**
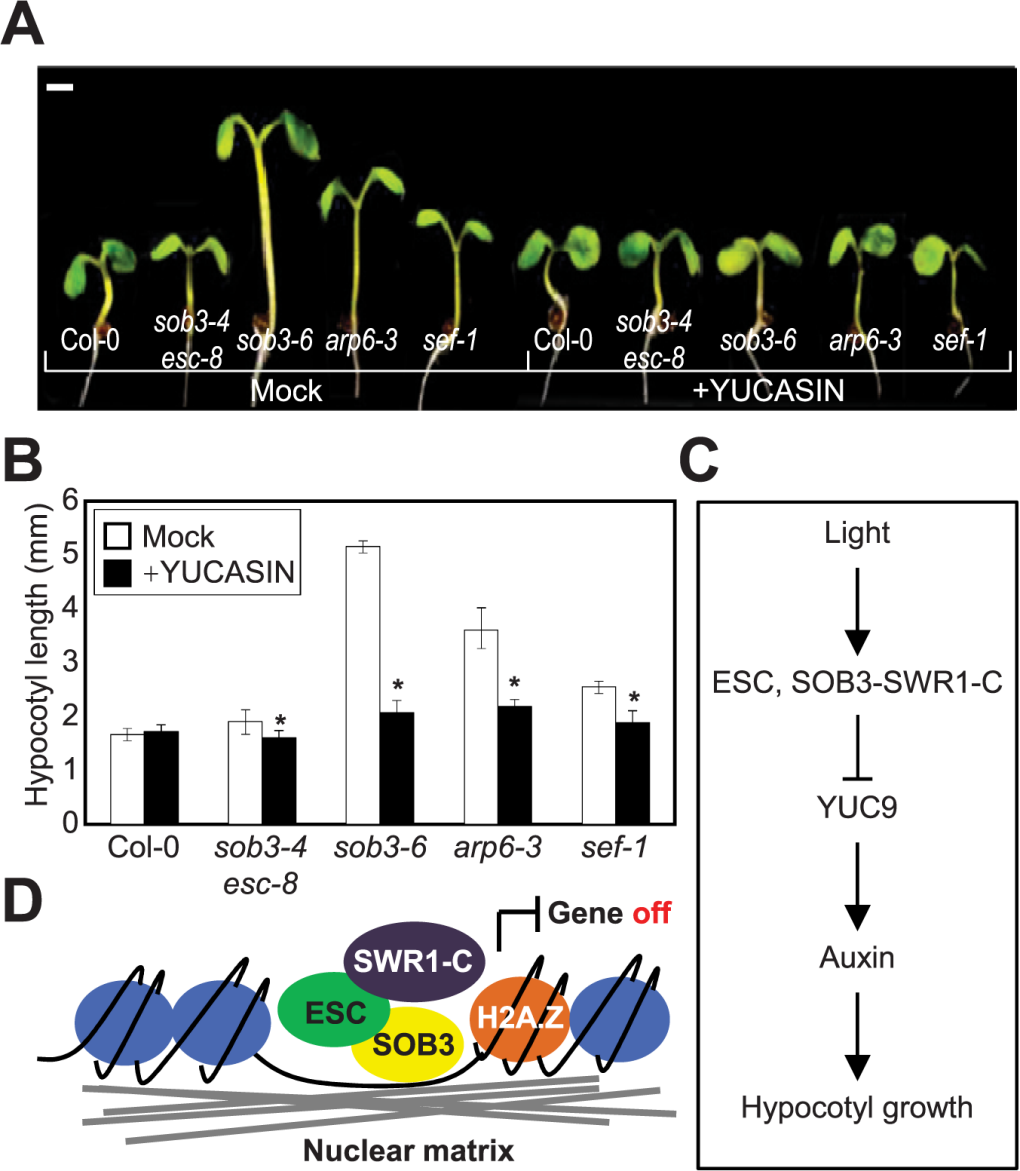
Genetic hierarchy of S/MAR-assisted H2A.Z exchange and auxin biosynthesis. (**A**) Effects of yucasin on hypocotyl elongation of *sob3-6*, *arp6-3* and *sef-1*. Seeds were germinated on MS medium supplemented with 250 uM yucasin, and incubated for 7 days under LD conditions. Scale bar, 1 mm. (**B**) Quantification of hypocotyl length. Hypocotyl lengths (*n* > 30 in each genotype) were measured using Image J applications (http://rsb.info.nih.gov/ij/). Three independent biological replicates were averaged, and the statistical significance of the measurements was determined by two-tailed Student's *t*-test assuming unequal variance (*P < 0.05). Bars indicate the standard error of the mean. (**C** and **D**) Proposed working diagram. Light-inducible ESC and SOB3 bind to the *YUC9* promoter and recruit the SWR1 complex that catalyzes H2A.Z exchange in order to suppress its expression. As a consequence, auxin biosynthesis is inhibited, and thus hypocotyl elongation is suppressed in light.

Since the AHLs affect *YUC9* expression, we wanted to test whether *YUC9* is genetically epistatic to ESC and SOB3 in the control of hypocotyl elongation. Generation of the triple mutant was unsuccessful, because all three *ESC*, *SOB3* and *YUC9* genes are on the same chromosome. As an alternative, we employed yucasin [42], a potent chemical inhibitor that specifically blocks the catalytic activities of YUCs, and evaluated its effects on the *sob3*-*4 esc*-*8* and *sob3-6* phenotypes. Similar to the auxin transport inhibitor, yucasin inhibited hypocotyl elongation of *sob3*-*4 esc*-*8* and *sob3-6* seedlings grown in light (Fig 5A and 5B), indicating that YUC-driven endogenous auxin biosynthesis is regulated by the AHL proteins in the control of hypocotyl elongation.

AHLs act with the ATP-dependent chromatin remodeling machinery. Our study proposed that H2A.Z exchange underlies auxin biosynthesis and thus hypocotyl elongation. To define the genetic association of H2A.Z replacement activity with auxin biosynthesis, we applied yucasin to the *sef-1* and *arp6-3* seedlings and measured hypocotyl length. Notably, the long hypocotyl phenotypes of *sef-1* and *arp6-3* mutants were restored by exogenous yucasin application (Fig 5A and 5B), demonstrating the functional coordination of SWR1 with S/MAR-binding proteins in auxin regulation of hypocotyl growth.

Taken together, the light-inducible S/MAR-binding proteins ESC and SOB3 suppress hypocotyl elongation in light-grown seedlings by reducing auxin biosynthesis. The AHL proteins bind directly to the *YUC9* promoter and facilitate the deposition of H2A.Z to repress gene expression. Thus, coordinated control of matrix attachment and chromatin modification underlies auxin biosynthesis and hypocotyl elongation (Fig 5C and 5D).

## Discussion

### AHL regulation of chromatin structure in *Arabidopsis*

Several lines of evidence suggest that S/MARs are closely associated with epigenetic regulation. Chromatin remodeling factors frequently bind to S/MAR sequences [43–45], and consistently, some S/MAR-binding proteins physically interact with chromatin modifiers to intricately control gene expression [27,28,46]. For example, the human S/MAR-binding protein SPECIAL AT-RICH SEQUENCE BINDING 1 (SATB1) recognizes and specifically binds to the base unpairing regions (BURs), a typical S/MAR sequence [47]. The diverse modes of SATB1 action are linked to different epigenetic modifications. For example, histone acetylation states at cognate regions are reversibly regulated depending on the phosphorylation status of SATB1 [48]. Phosphorylated SATB1 interacts with HISTONE DEACETYLASE 1 (HDAC1) and HDAC2 to stimulate closed chromatin formation, whereas the dephosphorylated protein allows interaction with histone acetyltransferase p300 to recruit regulatory proteins to target loci [48,49]. Also, SATB1 further regulates chromatin structure by employing the ATP-dependent chromatin remodeling factor IMITATION SWITCH (ISWI), which catalyzes H2A.Z exchange using energy derived from ATP hydrolysis [50,51]. The mouse S/MAR-binding protein SMAR1 is also known to regulate gene expression in connection with epigenetic regulation. SMAR1 interacts with the SIN3-HDAC1 complex to repress gene transcription [39,46].

Several studies have shown that some plant S/MARs are associated with chromatin modification, supporting the conservation of S/MAR-assisted molecular processes in higher eukaryotes. In *Arabidopsis*, the S/MAR-binding protein TRANSPOSABLE ELEMENT SILENCING VIA AT-HOOK (TEK)/AHL16 recruits a HDAC complex to TEs and TE-like repeat-containing loci and silences the chromatin regions [28]. In addition, this protein complex also regulates transcript accumulation of protein-coding genes including *FLOWERING LOCUS C* (*FLC*) and *FLOWERING WAGENINGEN* (*FWA*) to properly guide plant development. Furthermore, AHL22 represses *FT* expression by binding directly to AT-rich sequences in its promoter and recruits HDACs such as HDA6, HDA9, and HDA19 to ensure proper control of floral transition [27].

Here, we found a functional association between matrix attachment and ATP-dependent chromatin remodeling in *Arabidopsis*. The SOB3 protein interacts with the SWR1 complex. They are targeted to S/MARs in the *YUC9* promoter and suppress gene expression by exchanging the H2A.Z variant. H2A.Z is deposited in the *YUC9* promoter, whereas this deposition is impaired in the *sob3-6* mutant. Consistent with these results, the *sob3-6* dominant-negative mutant phenocopies genetic mutants of SWR1 components with regard to hypocotyl elongation in light, and the *YUC9* gene is specifically up-regulated in the mutants. Considering that yucasin suppresses the long hypocotyl phenotypes of *sob3-6*, *sef-1* and *arp6-3*, we conclude that matrix attachment and histone variant exchange are coordinated in the context of chromatin to properly regulate auxin biosynthesis and early seedling development.

### Regulation of auxin biosynthesis in hypocotyl elongation

Hypocotyl growth is primarily regulated by light. Light-dependent control of hypocotyl growth requires dense hormone metabolic networks [3–6]. In particular, auxin metabolism and signaling might account in part for light suppression of hypocotyl growth [11,13,16–18]. For instance, the basic leucine zipper (bZIP) transcription factor ELONGATED HYPOCOTYL 5 (HY5) is a central hub of light signaling pathways and regulates a large portion of light-responsive genes [52]. The HY5 protein promotes photomorphogenesis in light conditions, and consistently, the *hy5* mutants display long hypocotyl and other skotomorphogenic phenotypes [53]. It is noteworthy that auxin metabolism and signaling are significantly altered in *hy5* [18,54,55].

Additional molecular components are involved in regulating auxin biosynthesis during hypocotyl elongation. TCP4, a member of the TCP (TEOSINTE BRANCHED 1, CYCLOIDEA PROLIFERATING CELL FACTOR) family, is implicated in auxin regulation of hypocotyl growth. Transcript accumulation of *TCP4* correlates to cell elongation activities of seedlings in light. TCP4 directly activates *YUC5* and coordinates downstream auxin and brassinosteroid (BR) signaling [56]. The PHYTOCHROME-INTERACTING FACTOR 4 (PIF4) protein is also responsible for auxin-dependent hypocotyl growth. *PIF4*-overexpressing transgenic plants show increased hypocotyl length with high auxin accumulation [57]. PIF4 binds to and positively regulates the *YUC8* gene [57], contributing to hypocotyl elongation depending on light and temperature conditions. To date, the transcriptional regulation of auxin biosynthesis has been extensively demonstrated to be a key regulatory scheme for light-dependent hypocotyl growth.

We add another regulatory layer in the control of auxin biosynthesis and hypocotyl elongation. S/MAR-assisted ATP-dependent chromatin remodeling is important for light regulation of auxin biosynthesis. The light-inducible S/MAR-binding proteins ESC and SOB3 function in concert with the H2A.Z-exchanging enzyme to repress the *YUC9* gene in light. The AHL proteins specifically bind to a MAR region of the *YUC9* promoter and then recruit the SWR1 complex. H2A.Z deposition was enriched in the *YUC9* promoter in light-grown wild-type plants, but reduced in *sob3-6*, allowing higher Pol II recruitment. The SWR1 complex may also regulate other *YUC* expression. The SOB3 protein binds to the *YUC8* promoter and suppresses its expression [32]. SWR1 also binds weakly to the *YUC8* promoter in our condition and possibly influences transcript accumulation of *YUC8*. Possible scenario would be that the AHL-SWR1 complex regulates hypocotyl elongation by sequentially repressing *YUC8* and *YUC9* during early seedling development in light [32]. These observations indicate that epigenetic regulation adds complexity to auxin biosynthesis in the regulation of hypocotyl elongation.

Multiple *YUC* genes exist with redundant functions. Despite common biochemical activities, the upstream regulators are likely diverse. The large number of redundant genes not only compensates for impaired gene function but also establishes massive connections with various biological processes. Considering the huge signaling connections of auxin biosynthesis, hormone metabolism is intricately modulated through integration of multiple internal and external cues.

We cannot rule out the possibility that the S/MAR-binding proteins are associated with other hormone networks. Indeed, a recent study reports that SOB3 interacts with BR signaling in the control of hypocotyl elongation. SOB3-mediated auxin signaling converges with BR signaling to regulate *SAUR19* expression [58], which promotes hypocotyl growth. Given the myriad interaction networks of SOB3, extensive hormone signaling crosstalk may underlie SOB3-mediated hypocotyl elongation. Future works will further unravel elaborate coordination of multiple signaling pathways in the cell expansion process.

### Light-dependent histone variant exchange

H2A.Z is the most highly conserved histone variant, which is deposited by the SWR1 complex in eukaryotes [40,59]. *Arabidopsis* contains several homologs of SWR1 subunits, including PHOTOPERIOD-INDEPENDENT EARLY FLOWERING1 (PIE1), the only member that has ATPase activity, ARP4, ARP6, and SEF [40,59]. Since the genetic mutants of H2A.Z-exchanging machinery components share phenotypic changes, these components likely work together to regulate a variety of developmental processes such as hypocotyl growth, leaf development, flowering, and senescence [40,59,60].

Histone variant exchange is most likely associated with plant responses to ambient environments. The SWR1 complex is responsible for genome-wide changes in chromatin conformation and global transcriptional reprogramming upon ambient temperature changes [41]. In terms of flowering, chromatin conformation at the *FT* locus is significantly altered in a temperature-dependent manner by means of H2A.Z exchange [41]. Chromatin structure determines accessibility of transcriptional regulators, especially the transcriptional activator PIF4 [61]. In support of this, the *ARP6-*deficient mutants exhibit temperature-insensitive flowering phenotypes [41,61]. In addition, other temperature-dependent developmental changes, such as hypocotyl growth, are also influenced by SWR1-mediated ATP-dependent chromatin remodeling [41].

This study raises the possibility that the SWR1 complex is important for light regulation of developmental processes. Transcript accumulation of *ARP6* and *PIE1* is significantly elevated in light. Furthermore, long hypocotyl phenotypes of *ARP6*- and *SEF*-deficient mutants are observed in light conditions, while just mild phenotypic alterations are observed in seedlings grown in darkness. Taken together, the SWR1 complex might act as a gateway for crosstalk, integrating at least light and temperature signals. Environmental information is reflected in the context of chromatin. Chromatin structure might potentiate follow-up regulation and enable comprehensive signal transduction.

## Materials and methods

### Plant materials and growth conditions

*Arabidopsis thaliana* (Columbia-0 ecotype) was used for all experiments described, unless specified otherwise. Plants were grown under long day conditions (16-h light/8-h dark cycles) with cool white fluorescent light (100 umol photons m^-2^ s^-1^) at 23°C. The *arp6-3*, *sob3-4*, *sob3-4 esc-8*, *sob3-6* and *sob3-D* mutants were previously reported [31,35,60]. The *sef-1* mutant (SAIL_536_A05) [59] was obtained from the *Arabidopsis* Biological Resource Center (http://abrc.osu.edu/).

### Hypocotyl measurements

Seeds were plated on Murashige and Skoog medium (half-strength MS salts, 0.05% MES, pH 5.7, 1% sucrose, and 0.7% agar). Plates were stratified in darkness for 2 days at 4°C, exposed to white light for 6 h, and transferred to a culture room at 23°C under either long day or continuous dark conditions. Hypocotyl length was measured using the Image J application (http://rsb.info.nih.gov/ij/). 1-Naphthylphthalamic acid (NPA) was purchased from Sigma-Aldrich (N12507) (St. Louis, MO, USA), and 5-(4-chlorophenyl)-4H-1,2,4-triazole-3-thiol (yucasin) was obtained from Dr. Tomokazu Koshiba (Metropolitan University, Japan). NPA and yucasin were prepared in ethanol and DMSO, respectively, and used at final concentrations of 1 uM and 250 uM, respectively.

### Quantitative real-time RT–PCR analysis

Total RNA was extracted using the TRI agent (TAKARA Bio, http://www.takara-bio.com/). Reverse transcription (RT) was performed using Moloney murine leukemia virus (M-MLV) reverse transcriptase (Dr. Protein, http://www.doctorprotein.com) with oligo(dT18) to synthesize first-strand cDNA from 2 ug of total RNA. Complementary DNAs were diluted to 100 ul with TE buffer, and 1 ul of diluted cDNA was used for PCR amplification.

Quantitative RT-PCR reactions were performed in 96-well blocks using the Step-One Plus Real-Time PCR System (Applied Biosystems, https://www.appliedbiosystems.com/). PCR primers used are listed in S1 Table. The values for each set of primers were normalized relative to the *EUKARYOTIC TRANSLATION INITIATION FACTOR 4A1* (*eIF4A*) gene (At3g13920). All RT-qPCR reactions were performed in biological triplicates using total RNA samples extracted from three independent replicate samples. The comparative ΔΔCT method was employed to evaluate the relative quantities of each amplified product in the samples. The threshold cycle (CT) was automatically determined for each reaction by the system set with default parameters. Specificity of the RT-qPCR reactions was determined by melt curve analysis of amplified products using the standard method installed in the system.

### Measurement of GUS activity

Seven-day-old homozygous Col-0 x DR5-GUS and *sob3-6* x DR5-GUS seedlings grown under long day conditions were used for histochemical staining of GUS activity. Plant materials were immersed in 90% acetone for 20 min on ice, washed twice with rinsing solution [50 mM sodium phosphate pH 7.0, 0.5 mM K_3_Fe(CN)_6_, 0.5 mM K_4_Fe(CN)_6_], and subsequently incubated in staining solution containing 1mM 5-bromo-4-chloro-3-indolyl-D-glucuronide (X-Gluc) (Duchefa, Harlem, The Netherlands) at 37°C for 18–24 h.

### Chromatin immunoprecipitation (ChIP) assays

ChIP assays were performed as previously described [62]. To produce 35S:*MYC-SEF*, 35S:*MYC-ESC* and 35S:*MYC-SOB3* transgenic plants, a MYC-coding sequence was fused in frame to the 5′ end of the *SEF*, *ESC* and *SOB3* genes, and the gene fusions were subcloned under control of the Cauliflower mosaic virus (CaMV) 35S promoter. *Agrobacterium tumefaciens*-mediated *Arabidopsis* transformation was performed to produce transgenic plants.

Anti-MYC antibodies (05–724, Millipore, Billerica, MA, USA), anti-GFP antibodies (sc-9996, Santa Cruz Biotech., Dallas, Texas, USA), anti-Pol II (sc-33754, Santa Cruz, Dallas, Texas, USA), anti-H2A.Z (ab4174, Abcam, Cambridge, UK) antibodies and protein A/G agarose beads (sc-2003, Santa Cruz Biotech., Dallas, Texas, USA) were used for ChIP. DNA was purified using phenol/chloroform/isoamyl alcohol and sodium acetate (pH 5.2). Levels of precipitated DNA fragments were quantified by quantitative real-time PCR using specific primer sets (S2 Table). Values were normalized with respect to the level of input DNA. The values in control plants were set to 1 after normalization against *eIF4a* for quantitative PCR analysis.

### Y2H assays

Y2H assays were performed using the BD Matchmaker system (Clontech, http://www.clontech.com/). The pGADT7 vector was used for the GAL4 AD fusion, and the pGBKT7 vector was used for the GAL4 BD fusion. The PCR products were subcloned into pGBKT7 and pGADT7 vectors. The expression constructs were co-transformed into the yeast pJG69-4a strain harboring the LacZ and His reporter genes, and transformed cells were isolated by growth on selective medium. Interactions between two proteins were analyzed by growing on selective medium or measuring beta-galactosidase (b-Gal) activity using o-nitrophenyl-beta-D-galactopyranoside (ONPG) as the substrate.

### BiFC assays

The ARP4 gene was fused in-frame to the 5´ end of a gene sequence encoding the C-terminal half of EYFP in the pSATN-cEYFP-C1 vector (E3082). The *SOB3* cDNA sequence was fused in-frame to the 5´ end of a gene sequence encoding the N-terminal half of EYFP in the pSATN-nEYFP-C1 vector (E3081). The expression constructs were cotransformed into *Arabidopsis* protoplasts. YFP fluorescence was monitored by fluorescence microscopy using a Zeiss LSM510 confocal microscope (Carl Zeiss, Jena, Germany).

## Supporting information

S1 Fig Hypocotyl length of *sob3-4 esc-8* and *sob3-6* seedlings grown in darkness. Seeds were germinated and grown for 9 days on vertical MS medium in darkness. Hypocotyl lengths (*n* > 30 in each genotype) were measured using Image J applications (http://rsb.info.nih.gov/ij/). Biological triplicates were averaged. Bars indicate standard error of the mean. Scale bar, 1 mm.
(PDF)

S2 Fig Expression of *ESC* and *SOB3* in seedlings grown in continuous light or dark. Seeds were germinated and incubated in continuous light or dark for 9 days. Whole plants were harvested for total RNA isolation. Transcript accumulation was analyzed by quantitative RT-PCR (RT-qPCR). Biological triplicates were averaged and statistically analyzed by two-tailed Student's *t*-test assuming unequal variance (*P < 0.05). Bars indicate standard error of the mean.
(PDF)

S3 Fig Expression of hormone signaling genes in *sob3-6*. Ten-day-old seedlings grown under long-day conditions (LDs) were harvested for total RNA isolation. Transcript accumulation of hormone marker genes (**A**) and auxin transport genes (**B**) was analyzed by RT-qPCR. The *eIF4a* gene (At3g13920) was used as an internal control. Biological triplicates were averaged and statistically analyzed by two-tailed Student's *t*-test assuming unequal variance (*P < 0.05). Bars indicate standard error of the mean.
(PDF)

S4 Fig Kinetics of *YUC9* expression in *sob3-6*. Seedlings were grown under LD conditions for indicated time period (days). Transcript accumulation was analyzed by RT-qPCR. The *eIF4a* gene was used as an internal control. Biological triplicates were averaged and statistically analyzed by two-tailed Student's *t*-test assuming unequal variance (*P < 0.05). Bars indicate standard error of the mean. DAG, days after germination.
(PDF)

S5 Fig Hypocotyl length of 35S:*YUC9* transgenic seedlings grown in light. Seeds were germinated and grown for 9 days on vertical MS medium in LD conditions. Hypocotyl lengths (*n* > 30 in each genotype) were measured using Image J applications (http://rsb.info.nih.gov/ij/). Biological triplicates were averaged and statistically analyzed by two-tailed Student's *t*-test assuming unequal variance (*P < 0.05). Bars indicate standard error of the mean. Scale bar, 1 mm.
(PDF)

S6 Fig ChIP assays using antibody-free resin. Enrichment of the putative binding region (D region) of the *YUC9* promoter was analyzed by qPCR after ChIP with resin alone. Biological triplicates were averaged. Bars indicate standard error of the mean.
(PDF)

S7 Fig Binding of SOB3 and ESC to *YUC* promoters. Enrichment of putative binding regions in the *YUC* promoters was analyzed by ChIP-qPCR. We analyzed several different regions predicted by multiple webtools. Biological triplicates were averaged and statistically analyzed by two-tailed Student's *t*-test assuming unequal variance (*P < 0.05). Bars indicate the standard error of the mean.
(PDF)

S8 Fig beta-Galactosidase (b-Gal) activity assays. beta-Gal activity was quantified after growing yeast strains in liquid culture with *o*-nitrophenyl-beta-D-galactopyranoside as a substrate. Three independent measurements of b-Gal activities were averaged and statistically analyzed by two-tailed Student's *t*-test assuming unequal variance (*P < 0.05). Bars indicate standard error of the mean. Coexpression of GAL4 transcriptional activation domain (AD)-TPL and GAL4 DNA-binding domain (BD)-BES1 was performed as a positive control.
(PDF)

S9 Fig Yeast-two-hybrid (Y2H) assays. Y2H assays were performed with the SWR1 components fused with AD of GAL4 and the AHL proteins fused with the DNA BD of GAL4 for analysis of their interactions. Interactions were examined by cell growth on selective media. -LWHA indicates Leu, Trp, His, and Ade drop-out plates. -LW indicates Leu and Trp drop-out plates. GAL4 was used as a positive control.
(PDF)

S10 Fig Protein complex formation of SOB3 with SWR1. Partial fragments of YFP protein were fused with SOB3 and SEF. The constructs were transiently coexpressed in *Arabidopsis* protoplasts.
(PDF)

S11 Fig Hypocotyl length of genetic mutants of SWR1 components in darkness. Seeds were germinated and grown for 9 days on vertical MS medium in darkness. Hypocotyl lengths (*n* > 30 in each genotype) were measured using Image J applications (http://rsb.info.nih.gov/ij/). Biological triplicates were averaged. Bars indicate standard error of the mean. Scale bar, 1 mm.
(PDF)

S12 Fig Expression of genes encoding SWR1 components in seedlings grown in continuous light or continuous dark. Seeds were germinated and incubated in continuous light or continuous dark for 9 days. Whole plants were harvested for total RNA isolation. Transcript accumulation was analyzed by RT-qPCR. Biological triplicates were averaged and statistically analyzed by two-tailed Student's *t*-test assuming unequal variance (*P < 0.05). Bars indicate standard error of the mean.
(PDF)

S1 Table Primers used in RT-qPCR. RT-qPCR primers were designed using the Primer Express Software installed into the Applied Biosystems 7500 Real-Time PCR System. The sizes of PCR products ranged from 80 to 300 nucleotides in length. F, forward primer; R, reverse primer.
(PDF)

S2 Table Primers used in ChIP assays. The sizes of PCR products ranged from 80 to 300 nucleotides in length. F, forward primer; R, reverse primer.
(PDF)
