# Supplementary material for: Coordination of matrix attachment and ATP-dependent chromatin remodeling regulate auxin biosynthesis and Arabidopsis hypocotyl elongation
Source: PLoS One. 2017 Jul 26;12(7):e0181804. doi: 10.1371/journal.pone.0181804 (PMC5529009; doi:10.1371/journal.pone.0181804)
Supplement: S3 Fig — Ten-day-old seedlings grown under long-day conditions (LDs) were harvested for total RNA isolation. Transcript accumulation of hormone marker genes (A) and auxin transport genes (B) was analyzed by RT-qPCR. The eIF4a gene (At3g13920) was used as an internal control. Biological triplicates were averaged and statistically analyzed by two-tailed Student's t-test assuming unequal variance (*P < 0.05). Bars indicate standard error of the mean. (PDF) [file pone.0181804.s003.pdf]

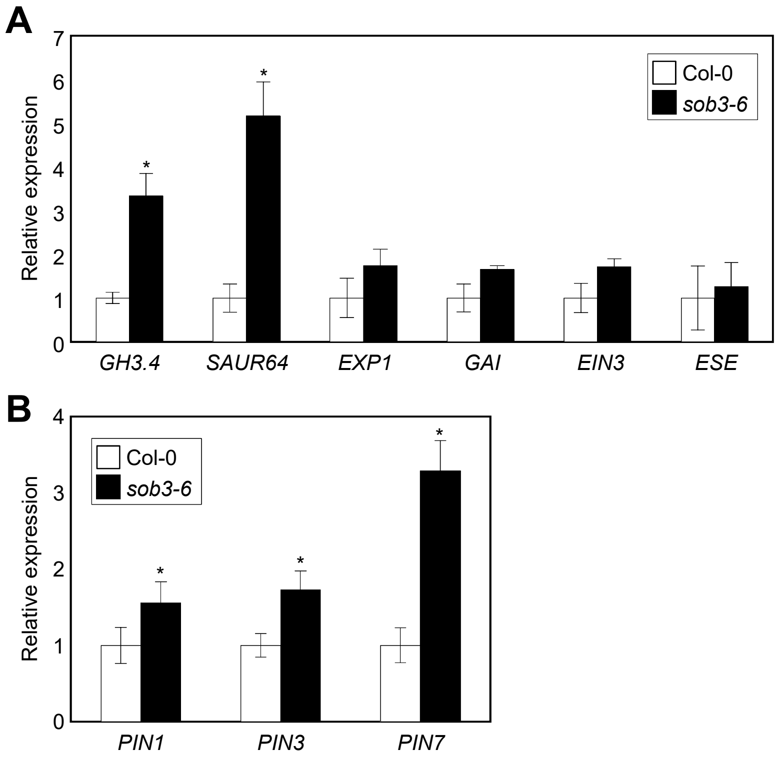


**S3 Fig. Expression of hormone signaling genes in *sob3-6*.**
Ten-day-old seedlings grown under long-day conditions (LDs) were harvested for total RNA isolation. Transcript accumulation of hormone marker genes (**A**) and auxin transport genes (**B**) was analyzed by RT-qPCR. The *eIF4a* gene (At3g13920) was used as an internal control. Biological triplicates were averaged and statistically analyzed by two-tailed Student's *t*-test assuming unequal variance (*$P < 0.05$). Bars indicate standard error of the mean.
