# Supplementary material for: Coordination of matrix attachment and ATP-dependent chromatin remodeling regulate auxin biosynthesis and Arabidopsis hypocotyl elongation
Source: PLoS One. 2017 Jul 26;12(7):e0181804. doi: 10.1371/journal.pone.0181804 (PMC5529009; doi:10.1371/journal.pone.0181804)
Supplement: S4 Fig — Seedlings were grown under LD conditions for indicated time period (days). Transcript accumulation was analyzed by RT-qPCR. The eIF4a gene was used as an internal control. Biological triplicates were averaged and statistically analyzed by two-tailed Student's t-test assuming unequal variance (*P < 0.05). Bars indicate standard error of the mean. DAG, days after germination. (PDF) [file pone.0181804.s004.pdf]

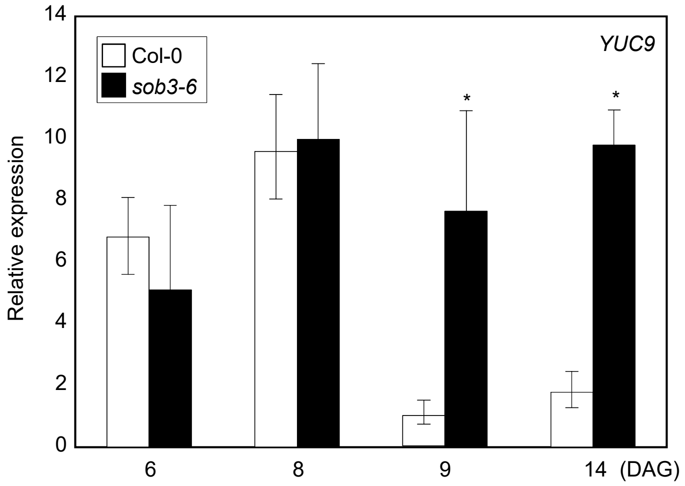

S4 Fig. Kinetics of *YUC9* expression in *sob3-6*.
Seedlings were grown under LD conditions for indicated time period (days). Transcript accumulation was analyzed by RT-qPCR. The *eIF4a* gene was used as an internal control. Biological triplicates were averaged and statistically analyzed by two-tailed Student's *t*-test assuming unequal variance (*$P < 0.05$). Bars indicate standard error of the mean. DAG, days after germination.
