# Supplementary material for: Coordination of matrix attachment and ATP-dependent chromatin remodeling regulate auxin biosynthesis and Arabidopsis hypocotyl elongation
Source: PLoS One. 2017 Jul 26;12(7):e0181804. doi: 10.1371/journal.pone.0181804 (PMC5529009; doi:10.1371/journal.pone.0181804)
Supplement: S5 Fig — Seeds were germinated and grown for 9 days on vertical MS medium in LD conditions. Hypocotyl lengths (n > 30 in each genotype) were measured using Image J applications (http://rsb.info.nih.gov/ij/). Biological triplicates were averaged and statistically analyzed by two-tailed Student's t-test assuming unequal variance (*P < 0.05). Bars indicate standard error of the mean. Scale bar, 1 mm. (PDF) [file pone.0181804.s005.pdf]

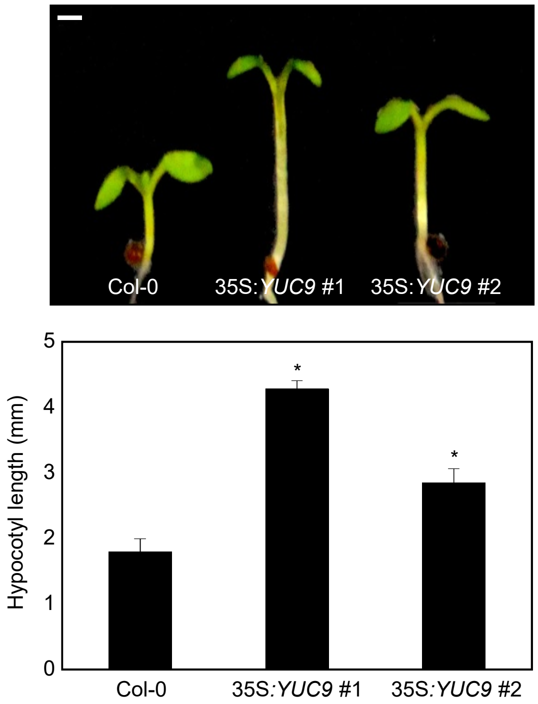

S5 Fig. Hypocotyl length of 35S:*YUC9* transgenic seedlings grown in light.
Seeds were germinated and grown for 9 days on vertical MS medium in LD conditions. Hypocotyl lengths (n > 30 in each genotype) were measured using Image J applications (http://rsb.info.nih.gov/ij/). Biological triplicates were averaged and statistically analyzed by two-tailed Student's t-test assuming unequal variance (*$P < 0.05$). Bars indicate standard error of the mean. Scale bar, 1 mm.
