# Supplementary material for: Coordination of matrix attachment and ATP-dependent chromatin remodeling regulate auxin biosynthesis and Arabidopsis hypocotyl elongation
Source: PLoS One. 2017 Jul 26;12(7):e0181804. doi: 10.1371/journal.pone.0181804 (PMC5529009; doi:10.1371/journal.pone.0181804)
Supplement: S6 Fig — Enrichment of the putative binding region (D region) of the YUC9 promoter was analyzed by qPCR after ChIP with resin alone. Biological triplicates were averaged. Bars indicate standard error of the mean. (PDF) [file pone.0181804.s006.pdf]

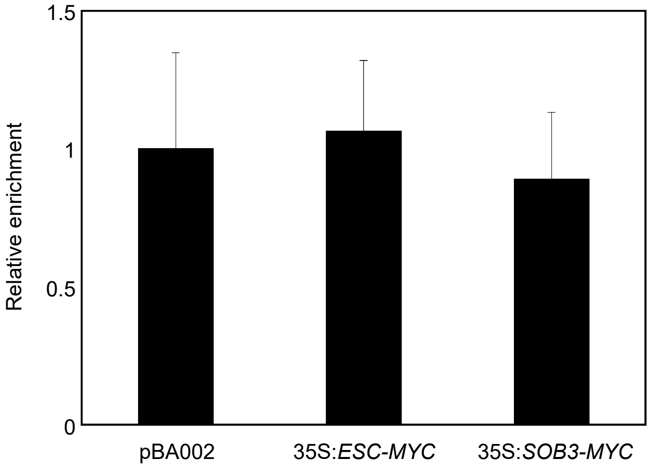

S6 Fig. ChIP assays using antibody-free resin.

Enrichment of the putative binding region (D region) of the *YUC9* promoter was analyzed by qPCR after ChIP with resin alone. Biological triplicates were averaged. Bars indicate standard error of the mean.
