# Supplementary figures and images for: Coordination of matrix attachment and ATP-dependent chromatin remodeling regulate auxin biosynthesis and Arabidopsis hypocotyl elongation
Source: PLoS One. 2017 Jul 26;12(7):e0181804. doi: 10.1371/journal.pone.0181804 (PMC5529009; doi:10.1371/journal.pone.0181804)

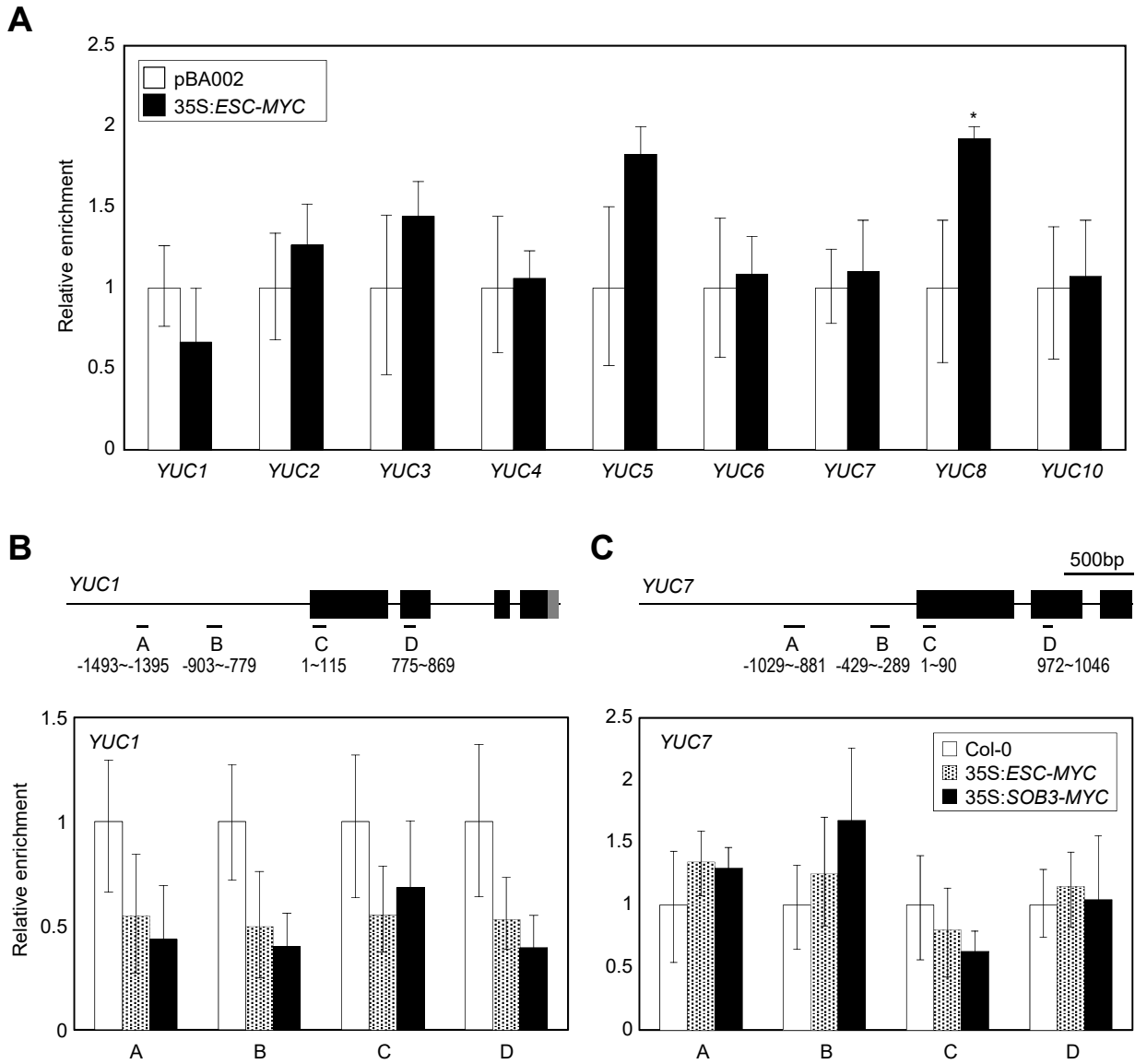

Supplement: S7 Fig — Enrichment of putative binding regions in the YUC promoters was analyzed by ChIP-qPCR. We analyzed several different regions predicted by multiple webtools. Biological triplicates were averaged and statistically analyzed by two-tailed Student's t-test assuming unequal variance (*P < 0.05). Bars indicate the standard error of the mean. (PDF) [file pone.0181804.s007.pdf]
