# Supplementary material for: Coordination of matrix attachment and ATP-dependent chromatin remodeling regulate auxin biosynthesis and Arabidopsis hypocotyl elongation
Source: PLoS One. 2017 Jul 26;12(7):e0181804. doi: 10.1371/journal.pone.0181804 (PMC5529009; doi:10.1371/journal.pone.0181804)
Supplement: S8 Fig — beta-Gal activity was quantified after growing yeast strains in liquid culture with o-nitrophenyl-beta-D-galactopyranoside as a substrate. Three independent measurements of b-Gal activities were averaged and statistically analyzed by two-tailed Student's t-test assuming unequal variance (*P < 0.05). Bars indicate standard error of the mean. Coexpression of GAL4 transcriptional activation domain (AD)-TPL and GAL4 DNA-binding domain (BD)-BES1 was performed as a positive control. (PDF) [file pone.0181804.s008.pdf]

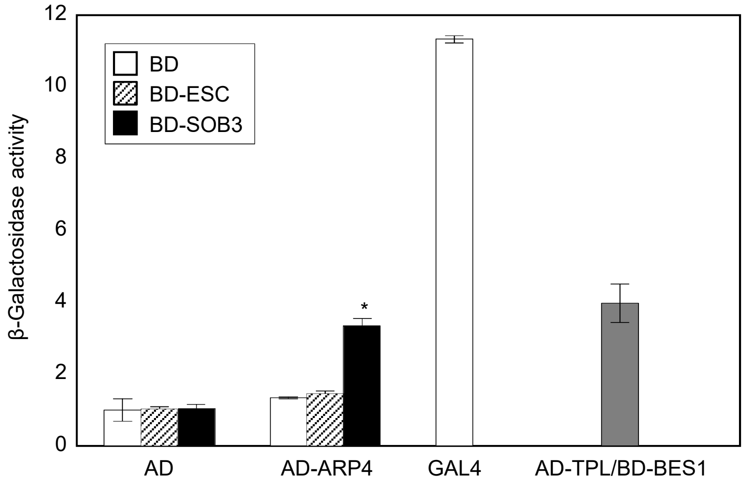

S8 Fig. β-Galactosidase (β-Gal) activity assays.

β-Gal activity was quantified after growing yeast strains in liquid culture with *o*-nitrophenyl-β-D-galactopyranoside as a substrate. Three independent measurements of β-Gal activities were averaged and statistically analyzed by two-tailed Student's *t*-test assuming unequal variance (*$P < 0.05$). Bars indicate standard error of the mean. Coexpression of GAL4 transcriptional activation domain (AD)-TPL and GAL4 DNA-binding domain (BD)-BES1 was performed as a positive control.
