# Supplementary material for: Coordination of matrix attachment and ATP-dependent chromatin remodeling regulate auxin biosynthesis and Arabidopsis hypocotyl elongation
Source: PLoS One. 2017 Jul 26;12(7):e0181804. doi: 10.1371/journal.pone.0181804 (PMC5529009; doi:10.1371/journal.pone.0181804)
Supplement: S9 Fig — Y2H assays were performed with the SWR1 components fused with AD of GAL4 and the AHL proteins fused with the DNA BD of GAL4 for analysis of their interactions. Interactions were examined by cell growth on selective media. -LWHA indicates Leu, Trp, His, and Ade drop-out plates. -LW indicates Leu and Trp drop-out plates. GAL4 was used as a positive control. (PDF) [file pone.0181804.s009.pdf]

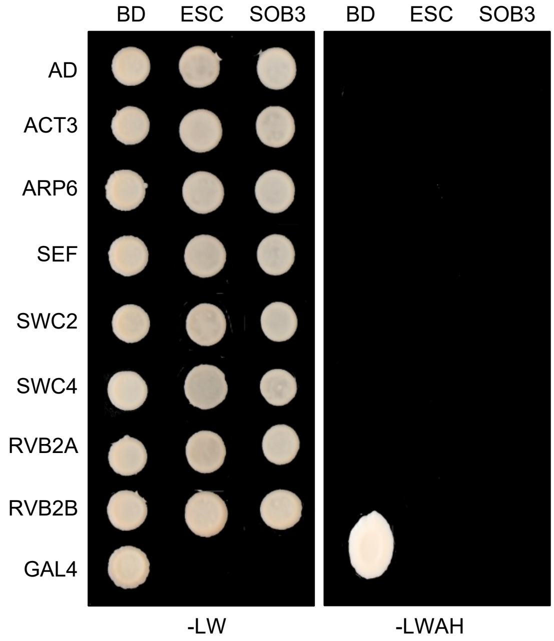

S9 Fig. Yeast-two-hybrid (Y2H) assays.

Y2H assays were performed with the SWR1 components fused with AD of GAL4 and the AHL proteins fused with the DNA BD of GAL4 for analysis of their interactions. Interactions were examined by cell growth on selective media. -LWHA indicates Leu, Trp, His, and Ade drop-out plates. -LW indicates Leu and Trp drop-out plates. GAL4 was used as a positive control.
