# Supplementary material for: Coordination of matrix attachment and ATP-dependent chromatin remodeling regulate auxin biosynthesis and Arabidopsis hypocotyl elongation
Source: PLoS One. 2017 Jul 26;12(7):e0181804. doi: 10.1371/journal.pone.0181804 (PMC5529009; doi:10.1371/journal.pone.0181804)
Supplement: S10 Fig — Partial fragments of YFP protein were fused with SOB3 and SEF. The constructs were transiently coexpressed in Arabidopsis protoplasts. (PDF) [file pone.0181804.s010.pdf]

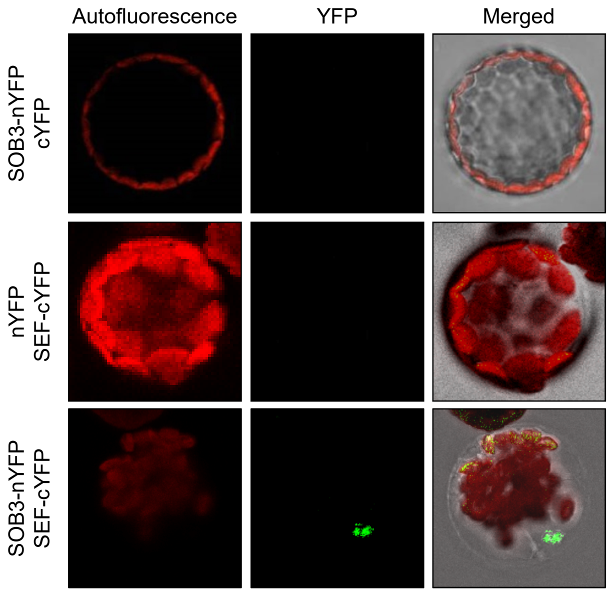

S10 Fig. Protein complex formation of SOB3 with SWR1.

Partial fragments of YFP protein were fused with SOB3 and SEF. The constructs were transiently coexpressed in *Arabidopsis* protoplasts.
