# Supplementary material for: Coordination of matrix attachment and ATP-dependent chromatin remodeling regulate auxin biosynthesis and Arabidopsis hypocotyl elongation
Source: PLoS One. 2017 Jul 26;12(7):e0181804. doi: 10.1371/journal.pone.0181804 (PMC5529009; doi:10.1371/journal.pone.0181804)
Supplement: S11 Fig — Seeds were germinated and grown for 9 days on vertical MS medium in darkness. Hypocotyl lengths (n > 30 in each genotype) were measured using Image J applications (http://rsb.info.nih.gov/ij/). Biological triplicates were averaged. Bars indicate standard error of the mean. Scale bar, 1 mm. (PDF) [file pone.0181804.s011.pdf]

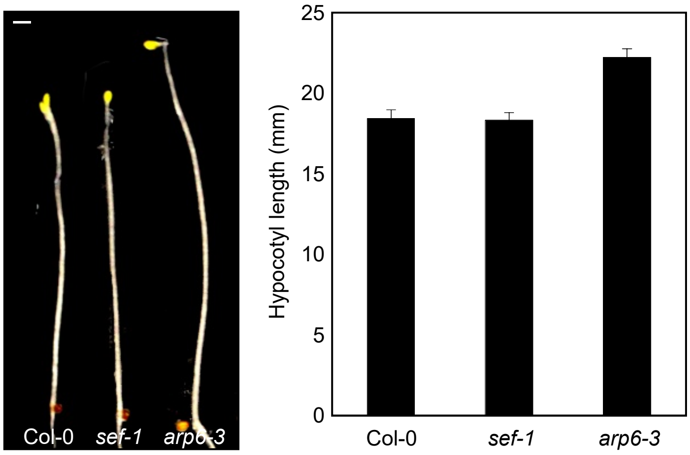

S11 Fig. Hypocotyl length of genetic mutants of SWR1 components in darkness.
Seeds were germinated and grown for 9 days on vertical MS medium in darkness. Hypocotyl lengths (*n* > 30 in each genotype) were measured using Image J applications (http://rsb.info.nih.gov/ij/). Biological triplicates were averaged. Bars indicate standard error of the mean. Scale bar, 1 mm.
