# Supplementary material for: Coordination of matrix attachment and ATP-dependent chromatin remodeling regulate auxin biosynthesis and Arabidopsis hypocotyl elongation
Source: PLoS One. 2017 Jul 26;12(7):e0181804. doi: 10.1371/journal.pone.0181804 (PMC5529009; doi:10.1371/journal.pone.0181804)
Supplement: S12 Fig — Seeds were germinated and incubated in continuous light or continuous dark for 9 days. Whole plants were harvested for total RNA isolation. Transcript accumulation was analyzed by RT-qPCR. Biological triplicates were averaged and statistically analyzed by two-tailed Student's t-test assuming unequal variance (*P < 0.05). Bars indicate standard error of the mean. (PDF) [file pone.0181804.s012.pdf]

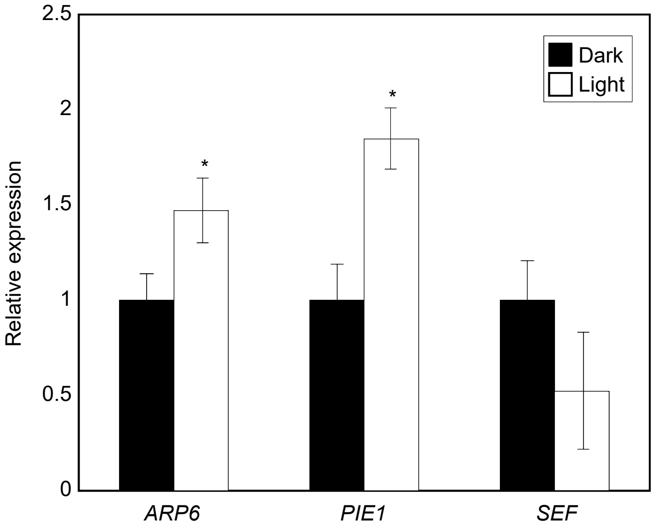

S12 Fig. Expression of genes encoding SWR1 components in seedlings grown in continuous light or continuous dark.

Seeds were germinated and incubated in continuous light or continuous dark for 9 days. Whole plants were harvested for total RNA isolation. Transcript accumulation was analyzed by RT-qPCR. Biological triplicates were averaged and statistically analyzed by two-tailed Student's *t*-test assuming unequal variance (*$P < 0.05$). Bars indicate standard error of the mean.
