# Supplementary material for: Coordination of matrix attachment and ATP-dependent chromatin remodeling regulate auxin biosynthesis and Arabidopsis hypocotyl elongation
Source: PLoS One. 2017 Jul 26;12(7):e0181804. doi: 10.1371/journal.pone.0181804 (PMC5529009; doi:10.1371/journal.pone.0181804)
Supplement: S1 Table — RT-qPCR primers were designed using the Primer Express Software installed into the Applied Biosystems 7500 Real-Time PCR System. The sizes of PCR products ranged from 80 to 300 nucleotides in length. F, forward primer; R, reverse primer. (PDF) [file pone.0181804.s013.pdf]

Primer	Usage	Sequence	
		F	R
eIF4a	RT-qPCR	TGACCACACAGTCTCTGCAA	ACCAGGGAGACTTGTTGGAC
ARP6	RT-qPCR	ACGAAATCGACCTCACCTCC	CGGAAGGGATTGAGAGTGGT
CYP79B2	RT-qPCR	CTTACCGCCGATGAAATCAA	TTGTGTCAGAAAGTGCCACG
CYP79B3	RT-qPCR	AACAAGACGCACTCTTTGCG	GCCTCTTGATTGCATTTCCA
EIN3	RT-qPCR	GCGTTGATGCAACACTGTGA	GTTGAGGCCACCAATCCTCT
ESE1	RT-qPCR	GGACGTTTAACACAGCGGAA	ATATCCCGACGACGAGGAAG
ESC	RT-qPCR	ATATTTTTAGCCGGAGGGCA	TCTCTCGAAAACCGCATTTG
EXP1	RT-qPCR	TCTTACCTTAACGGACAATC	GAAGCACCACTTCTTTTTAG
GAI	RT-qPCR	CTGGTTGACTCGCAGGAGAA	TTTCTCATCGCTCCGATTTG
GH3.4	RT-qPCR	TTCAATCTGGGATGGCTTCA	CACCGGAACTTTGCTCTTGA
PIE1	RT-qPCR	GAATCTAAGGATCGGGCAGC	CCAGCAAGTAGGTGAAGGCA
PIN1	RT-qPCR	CACCTGGTCCCTCATTTCCT	TGCCTGGATAATGGCAACAT
PIN3	RT-qPCR	TTTCCCTCTCCACACTTCCC	CACTGGAGGACGACGATTTG
PIN7	RT-qPCR	ATGCTGGTCTTGGTATGGCA	GGCAATGCAGCTTGAACAAT
SAUR64	RT-qPCR	AAGAAGAGTTTGGCCTCCCA	CTGCATCCATTCGTCTTTGG
SEF	RT-qPCR	CGGCTCTTACAAGCAACGAC	AGTCGGTACATGCGACGGTA
SOB3	RT-qPCR	AGAGATAGCCCCAACGTGCT	GGCAACACCGTACCTGTGAG
TAA1	RT-qPCR	ACTCGAGGAAACCCGAAAAA	TCACCGTACACCTGTCACCC
YUCCA1	RT-qPCR	TCCTAACGGCTGGAGAGGAG	GTGGACCCCTTGATTTCGTC
YUCCA2	RT-qPCR	GAGAATGCCGAGGAGGTGAT	CAAACTTCCATCCCGGAGTT
YUCCA3	RT-qPCR	GTTTCAAAACCTCATCCCCG	GCAATGCAGTTAGCTCGCTC
YUCCA4	RT-qPCR	GCGAGCTTCCTCTTATGCCT	CGAACTCTGCTTTCTCGACG
YUCCA5	RT-qPCR	TCACAACGGAGTTTGAAGGC	AGACTTCCATGCCGGAGTTT
YUCCA6	RT-qPCR	CTACGCTCGGAGGTTTGACA	ACTCCGTCGTGCCTTCTTCT
YUCCA7	RT-qPCR	TCAATGGTCGTTCGAAGCTC	TACCTGCGGTGTTCTTCAGC
YUCCA8	RT-qPCR	TGAGTTTTCCGGTGAGGTGA	AGAAACTTCCATGCCGGAGT
YUCCA9	RT-qPCR	GTTCTTGTCGTCGGATGTGG	CCCCATAATCTCCCTCGGTA
YUCCA10	RT-qPCR	AGGTGGAAATTCCGGTATGG	ATGACAGGAGCTTTTCCGGT
YUCCA11	RT-qPCR	TGAACAAGCTAATCACCGGC	TCTTCCCCTTCCAGTGATCC

S1 Table. Primers used in RT-qPCR.
RT-qPCR primers were designed using the Primer Express Software installed into the Applied Biosystems 7500 Real-Time PCR System. The sizes of PCR products ranged from 80 to 300 nucleotides in length. F, forward primer; R, reverse primer.
