# Supplementary material for: Coordination of matrix attachment and ATP-dependent chromatin remodeling regulate auxin biosynthesis and Arabidopsis hypocotyl elongation
Source: PLoS One. 2017 Jul 26;12(7):e0181804. doi: 10.1371/journal.pone.0181804 (PMC5529009; doi:10.1371/journal.pone.0181804)
Supplement: S2 Table — The sizes of PCR products ranged from 80 to 300 nucleotides in length. F, forward primer; R, reverse primer. (PDF) [file pone.0181804.s014.pdf]

	Sequence	
**Primer**	**F**	**R**
eIF4a	TGACCACACAGTCTCTGCAA	ACCAGGGAGACTTGTTGGAC
YUCCA1	TTTCAAAATATCCAGGCAAT	AGTAAAATGGCAGTCCAAAA
YUCCA2	ACACTCGAGAAATGCAAAAT	AAAAGAAACCAAGCTGAAAA
YUCCA3	ATGCAAAGAAAAGAAGATGC	TTGATGCAAATTGGTAATTG
YUCCA4	ATCATGGAAGTCATGAAACA	TTTGATACTTTCGTGAGCAA
YUCCA5	TCATTATGATTGGTCCATTG	TGGAATCATTCGATTCTTTT
YUCCA6	ATTGCACCCATTTCTCTAAA	CATCAAACTAAGCATCGAAA
YUCCA7	TAGATTTGGTTTGTGCATTG	GGTTTTAATCCCGTTCATTA
YUCCA8	TTCCGTACCTAAAAATTGGA	AAGAAAAAGATGACGTGGAA
YUCCA10	TGTATTCAAACCCCATTCTT	ATCTTGAAAATGTGAACACG
YUCCA1(A)	ATGTTTGAGTTGGAAACGTCTG	AAAAATAGCCGAAGGTGATTATAAAC
YUCCA1(B)	TGTAGGTTTTTTTACACAAATATAG	TCTGAGACATTCATGTTTAACCTATAG
YUCCA1(C)	ATGGAGTCTCATCCTCACAAC	TCGAGAGACATGCTGAAGTG
YUCCA1(D)	ATCAACATTTGGAGTTGGAATG	TTCCAAACGAAAGATTGG
YUCCA7(A)	CAATAATACAAGAGGAATAATTGTG	GTGAAATCTATTCATGTGCAG
YUCCA7(B)	CAAATCATTCGGATACATCATAC	AGAATGAAACTGACACTTATTGAAAAG
YUCCA7(C)	ATGTGTAATAACAATAACACAAGTTGTG	ATTCACCCAGATGCAACGAC
YUCCA7(D)	AAGAGGCCTGAAATTGGACC	AATGGCTCCGATGTCTAGC
YUCCA9(A)	TTTGATCTAAGTCTTTATATATAATTTCATATTCG	AATATTTTTGGGAAGTACAGTATATATGC
YUCCA9(B)	ATTAACTCGGAGATATTAAATGATTTCTATAGGTTAG	GTTTCCTTTTACTTTTATTTTTACTTTAAGACTAAC
YUCCA9(C)	ACTCTCTAGTTTCTCTACAATGGTCGTTTAG	TCCAGTACCATGAGCTTCAGTATGATC
YUCCA9(D)	AATCGAATATTTTTATTTTTTTATACAAAAAAACG	TTTAGTTAGGACCTAACTTTTGTCATATGC
YUCCA9(E)	TTGTGGTCGAGAGATCAGATTG	AATTTCTTGGGCAAGTGAAG
YUCCA9(F)	TTATCAAACTATGGGCTTAAAAG	TTCTACGTCGCCGGATTTTATC

S2 Table. Primers used in ChIP assays.

The sizes of PCR products ranged from 80 to 300 nucleotides in length. F, forward primer; R, reverse primer.
